# *In silico* evaluation of bioactive compounds from the sea urchin *Temnopleurus toreumaticus*: Potential multifunctional agents against mosquito vectors and tropical pathogens

**DOI:** 10.1371/journal.pone.0341080

**Published:** 2026-01-23

**Authors:** Karnan Ramachandran, Senthil Bakthavatchalam, Velavan Sivanandham, Shunmuga Vadivu Ramalingam, Pitchaimuthu Mariappan, Renganathan Senthil, Chandramohan Govindasamy, Khalid S. Al-Numair, Ramachandran Vinayagam, Zhi-Hong Wen, Hsien-Kuo Chin

**Affiliations:** 1 Harman Institute of Science Education and Research, Thanjavur, Tamil Nadu, India; 2 Department of Chemistry, Faculty of Engineering and Technology, SRM Institute of Science and Technology, Chennai, Tamil Nadu, India; 3 Department of Biochemistry, SRM Dental College, Ramapuram, Tamil Nadu, India; 4 PG and Research Department of Zoology, Rajah Serfoji Government College (Autonomous), Thanjavur, Tamil Nadu, India; 5 Department of Bioinformatics, School of Lifesciences, Vels Institute of Science Technology and Advanced Studies (VISTAS), Pallavaram, Tamil Nadu, India; 6 Department of Community Health Sciences, College of Applied Medical Sciences, King Saud University, Riyadh, Saudi Arabia; 7 Department of Biotechnology, Institute of Biotechnology, College of Life and Applied Sciences, Yeungnam University, Daehak-Ro, Republic of Korea; 8 Department of Marine Biotechnology and Resources, National Sun Yat-Sen University, Kaohsiung, Taiwan; 9 National Museum of Marine Biology & Aquarium, Pingtung, Taiwan; 10 Division of Cardiovascular Surgery, Department of Surgery, Kaohsiung Armed Forces General Hospital, Kaohsiung, Taiwan; 11 Institute of Medical Science and Technology, National Sun Yat-sen University, Kaohsiung, Taiwan; 12 National Defense Medical University, Taipei, Taiwan; Guru Nanak College, INDIA

## Abstract

This study investigates the ethanolic extract of the sea urchin *Temnopleurus toreumaticus* (test and spine) for its larvicidal efficacy against *Aedes aegypti*, cytotoxicity, and *in silico* interactions supporting potential antimalarial and antiviral activities. Zoochemical profiling by Fourier-transform infrared spectroscopy (FT-IR) showed the presence of functional groups, while gas chromatography-mass spectrometry (GC-MS) analysis identified 24 bioactive compounds with insecticidal and enzyme-inhibitory properties. The extract exhibited strong larvicidal (LC₅₀ = 164.18 µg/mL) and pupicidal (LC₅₀ = 209.91 µg/mL) effects on *A. aegypti* in a concentration‑dependent manner, supported by significant acetylcholinesterase inhibition (IC₅₀ = 151.49 µg/mL). Morphological examinations showed epithelial disorganization and structural damage in treated larvae and pupae. *In silico* docking confirmed that the identified zoochemicals exhibited binding affinities ranging from –7 to –8 kcal/mol for juvenile hormone binding protein (PDB 5V13), –5 to –6 kcal/mol for acetylcholinesterase (PDB 1DX4), –4 to –5 kcal/mol for *Plasmodium falciparum* lactate dehydrogenase (PDB 1CEQ), and –4 to –5 kcal/mol for chikungunya nsP2 protease (PDB 3TRK), indicating their multitarget larvicidal, antimalarial, and antiviral potential. Yeast cell-based assays indicated cytotoxic activity (EC₅₀ = 159.27 µg/mL) with a strong dose-response relationship. Overall, the ethanolic extract of *T. toreumaticus* is a promising lead for next‑generation, environmentally safe biocontrol agents targeting vector‑borne diseases such as malaria and chikungunya, while clearly emphasizing that the antiviral and antimalarial properties are currently supported only by *in silico* evidence and require further *in vitro* and *in vivo* validation.

## Introduction

Vector-borne diseases transmitted by *Aedes aegypti* and *Anopheles* mosquitoes continue to pose a significant global health burden, affecting hundreds of millions of people annually [1,2]. Mosquito control remains the cornerstone of vector management; however, the excessive reliance on synthetic insecticides has resulted in environmental toxicity, emergence of resistance, and threats to non-target organisms [3,4]. Consequently, there is increasing scientific interest in developing eco-friendly biocontrol strategies derived from natural sources.

Marine organisms, in particular echinoderms such as sea urchins, represent a promising yet underexplored source of bioactive compounds with potential mosquitocidal properties [5–7]. Sea urchins (Phylum Echinodermata) are benthic invertebrates known for their biochemical diversity and capacity to produce nitrogenous, phenolic, and terpenoid metabolites. Previous reports on species including *Salmacis virgulata*, *Diadema savignyi*, and *Echinometra mathaei* have demonstrated antioxidant, anticancer, and insecticidal activities, underscoring the untapped potential of echinoid extracts [6–8].

Marine natural products have historically contributed to pharmaceutical innovation by providing structurally unique molecules for therapeutic applications. Despite the global diversity of sea urchins, encompassing more than 800 species, the zoochemical properties of many Indian echinoids remain unexplored [9,10]. One such species, *Temnopleurus toreumaticus,* a black-spined regular sea urchin commonly distributed along the south-eastern Indian coast, is often discarded as marine waste. Its calcareous spines and tests contain complex organic and inorganic matrices, which may harbor potent bioactive molecules. However, no previous studies have systematically investigated the larvicidal, pupicidal, or enzyme-inhibitory potential of *T. toreumaticus* in mosquito vector control [11,12].

The spines and tests of sea urchins are rich in proteins, minerals, and polysaccharides, and have been consumed traditionally for their nutritional and medicinal values. In traditional Asian medicine, sea urchin-derived materials have been used to alleviate inflammation and other metabolic conditions [13]. Modern investigations further suggest that bioactive compounds from echinoids exhibit antimicrobial, antitumor, antioxidant, and antiviral activities, with composition varying across species depending on diet and habitat [14].

Recent studies on related echinoderm species such as the sea cucumber *Bohadschia cousteaui* and sea urchin *Anthocidaris crassispina* have revealed diverse bioactive metabolites with insecticidal, antioxidant, and antimicrobial properties [15–18]. Echinoid extracts have identified as containing phenolic and fatty acid derivatives possessing strong radical scavenging and cytotoxic capabilities. These findings collectively highlight the pharmacological potential of echinoderms and reinforce the need to explore underutilized marine species for sustainable biotechnological applications.

The present study aims to investigate the larvicidal, pupicidal, and acetylcholinesterase inhibitory activities of ethanolic extracts derived from the spines and tests of *T. toreumaticus*. GC-MS and FT-IR analyses were performed to characterize the associated bioactive compounds, followed by *in silico* modeling to elucidate their molecular interactions and potential antiviral and antimalarial relevance. This integrated approach not only identifies *T. toreumaticus* as a novel source of natural insecticidal agents but also supports its potential role in sustainable marine biowaste valorization and vector control.

## Materials and methods

### Collection and observation of the sea urchin shell-like test

**Fig 1:**
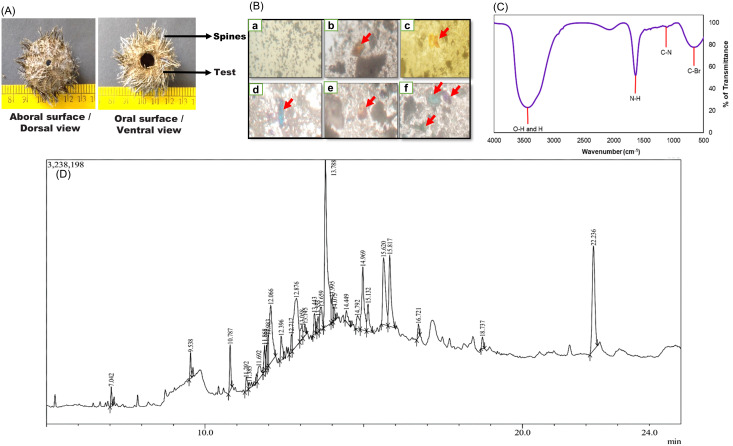
Morphology and chemical characterization of *T.toreumaticus.* **(A)** Test and spines of the sea urchin *T.toreumaticus* (Leske, 1778). **(B)** Histochemical screening of *T. toreumaticus* test and spines showing (a) control, (b) alkaloids, (c) terpenoids, (d-f) polyphenol. **(C)** FT-IR Spectrum of the ethanolic extract of *T. toreumaticus* test and spines. **(D)** GC-MS chromatogram of *T. toreumaticus* ethanolic extract.

Dead and dried sea urchin *Temnopleurus toreumaticus* (Voucher number: RSGC-Z-K02; Fig 1A) were collected from the southeastern coast of Mallipattinam (10.2806° N, 79.3170° E), Thanjavur district, Tamil Nadu, India. *T. toreumaticus* was taxonomically identified based on morphological characteristics [19]. *T. toreumaticus* were deposited at the Zoological Museum, PG and Research Department of Zoology, Rajah Serfoji Government College, Tamil Nadu, India.

### Zoochemical extraction and screening

Ten ununiformed, dead, and dried sea urchins (*T. toreumaticus*), with black spines intact, were crushed into a fine homogenized powder using a mortar and pestle. A 10 g portion of the powdered material was mixed with 100 mL of ethanol (99.5%) and heated at 50–60 °C for 10 minutes. The mixture was then kept in the dark for 48 hours. After 48 hours of extraction, the ethanolic sea urchin extract was filtered through Whatman No. 1 filter paper. The filtrate was then concentrated using a water bath maintained at 40–50 °C, and a paste-like extract was obtained. The resulting ethanolic zoo extract was initially subjected to zoochemical profiling, followed by characterization using Fourier-transform infrared spectroscopy (FT-IR) and gas chromatography-mass spectrometry (GC-MS).

### FT-IR and GC-MS analysis

FT-IR spectroscopic analysis was conducted to identify the functional groups present in the ethanolic extract of *T. toreumaticus*. The measurements were performed using an FT-IR spectrophotometer operating in the mid-infrared region between 4000 and 400 cm^-1^. Each spectrum was obtained as an average of 50 scans to improve the signal-to-noise ratio and ensure reliable detection of characteristic absorption bands corresponding to different structural moieties within the extract [20].

GC-MS analysis was performed using a Shimadzu GC-MS-QP2010 Plus system equipped with an AOC-20i autosampler and interfaced to a mass selective detector. Separation was achieved on an RTX-5MS capillary column (30 m length, 0.32 mm internal diameter, and 0.50 µm film thickness). The instrument was operated in electron impact (EI) ionization mode at 70eV. Helium (99.99%) served as the carrier gas, maintained at a constant flow rate of 1.73 mL/min. A 5 µL aliquot of the sample was injected in split mode (10:1) with an injector temperature of 270 °C and an ion source temperature of 200 °C.

The oven temperature was initially programmed at 40 °C (held for 2 min), then ramped at 8 °C/min to 150 °C, further increased at 8 °C/min to 250 °C, and finally held isothermally at 280 °C for 20 min. The total chromatographic run time was 51.25 min. Mass spectra were acquired in the range of 40–450 Da at a scan rate of 0.5 s per spectrum. Data acquisition and processing were performed using TurboMass software (version 5.2.0). Compounds were identified based on their mass spectral patterns by comparison with reference spectra available in the NIST and Wiley libraries. Retention indices were computed and evaluated for confirmation of compound identities. Relative percentage composition of each component was determined from the ratio of its individual peak area to the total chromatographic peak area [21].

### Histochemical examination

Histochemical analysis was conducted on the test and spine powders of *T. toreumaticus* using specific reagents widely recommended in the literature for detecting key secondary metabolites. This qualitative method provides a rapid and preliminary confirmation of the presence of important zoochemicals in this sea urchin. The histochemical techniques to characterize the metabolite profile of *T. toreumaticus* test and spine powder, thereby filling a critical gap in understanding its biochemical composition.

#### Test for polyphenol.

Approximately 0.1 g of sea urchin powder was placed in the cavity of a glass slide (2 × 7 cm) and completely covered. Subsequently, 3–5 drops of toluidine blue reagent (0.01%) were added, and the sample was allowed to react at room temperature for 30 min. After incubation, the slide was examined under a light microscope. The appearance of blue, green, and red coloration indicated the presence of polyphenolic compounds [22].

#### Test for terpenoids.

Approximately 0.1 g of sea urchin powder was placed in the cavity of a glass slide (2 × 7 cm) and completely covered. Then, 3–5 drops of 2,4-dinitrophenylhydrazine (DNPH) reagent (0.3% w/v) were added, and the slide was allowed to stand at room temperature for 30 min. After incubation, the slide was examined under a light microscope. The appearance of an orange coloration indicated the presence of terpenoid compounds [23].

#### Test for alkaloids.

A glass slide (2 × 7 cm) cavity was filled with 0.1 g of sea urchin powder and fully covered. Then, 3–5 drops of Wagner’s reagent (2 g iodine and 6 g potassium iodide in 100 mL H_2_O) were added, and the slide was left at room temperature for 30 min. Examination under a light microscope revealed a reddish-brown coloration, indicating the presence of alkaloid compounds [23].

### Larvicidal and pupicidal activity

#### Rearing of larvae.

Wild *A. aegypti* (Linnaeus, 1762) larvae were collected from Kathattipatti village (10.71284° N, 78.91580° E), Sengipatti, Thanjavur District, Tamil Nadu, India. The collected third instar larvae were maintained under controlled laboratory conditions at 27 °C, 75 ± 5% relative humidity, and a 14 h light–dark photoperiod until they developed into fourth instar larvae and pupae. During rearing, the larvae were provided with a feeding solution containing 6% glucose (60 g/L) and yeast powder at a concentration of 0.05 g/L [24–26] with minor modifications [27]. The resulting fourth instar larvae and pupae were then used in larvicidal and pupicidal bioassays.

#### Bioassay.

The ethanolic extract derived from the test and spines of *T. toreumaticus* was evaluated for its larvicidal and pupicidal efficacy against 4^th^ instar larvae and pupa of *A. aegypti*. The bioassay was conducted following the standard protocol recommended [28]. *A. aegypti* larvae and pupa were placed in containers filled with microbe-free deionized water. Test solutions of *T. toreumaticus* ethanolic extract were prepared at various concentrations (50, 100, 150, 200, and 250 µg/mL), each in a final volume of 100 mL, using deionized water as the solvent in 250 mL glass beakers. Malathion (1–5 µg/mL) was employed as the positive control for comparison in the larvicidal and pupicidal bioassays. Each bioassay was performed separately for each concentration in larvicidal and pupicidal activity. Twenty larvae and pupa were introduced into each beaker containing the test solution, while a control group, devoid of the extract, was maintained under identical conditions to assess natural mortality [29]. All experiments were conducted in triplicate to ensure reproducibility. Larval and pupa mortality was recorded after 24 h of exposure, following the procedure outlined [30].

### *In vitro* AChE inhibition assay

#### Mosquito larvae AChE enzyme extraction.

The extraction of the insect acetylcholinesterase (AChE) enzyme was carried out with slight modification. Mosquito larval heads were separated from their bodies using a 1 mm thick, 5 cm long stainless-steel needle under a dissection microscope at 20 × magnification. The larvae were homogenized to prepare a 5% (w/v) homogenate using a solution containing 1 mL of Triton X-100, 38.03 mg of ethylene glycol tetraacetic acid (EGTA), 5.845 g of NaCl, and 80 mL of Tris buffer (10 mM, pH 7.0). The homogenate was then centrifuged at 5000 rpm for 5 min (4–6 °C), and the resulting supernatant was collected for acetylcholinesterase inhibition assays [31].

#### AChE inhibition assay.

The AChE inhibition assay was performed following the method [32,33], with slight modifications. A 50 μL aliquot of the AChE enzyme supernatant was transferred into each cuvette, followed by the addition of 100 μL of *T. toreumaticus* ethanolic extract at varying concentrations (50, 100, 150, 200, and 250 μg/mL). Subsequently, 750 μL of phosphate buffer (maintained at 1–4 °C) was added to each reaction mixture. The mixtures were incubated for 10 minutes at 25 °C. After incubation, 100 μL of 0.4 mM acetylthiocholine iodide and 0.3 mM DTNB (5,5′-dithiobis-(2-nitrobenzoic acid)) were introduced to initiate the enzymatic reaction. The reaction was allowed to proceed for 30 minutes at room temperature, during which a yellow or colorless change in the solution was noted, indicating enzyme activity. Absorbance was measured at 412 nm using a spectrophotometer, and the percentage of enzyme inhibition was calculated as described [34].


$$\text{AChE}\;\text{inhibition}\;(\%)=\left({\text{A}}_{\text{C}}-{\text{A}}_{\text{S}}\right)/{\text{A}}_{\text{C}}\times \text{100}$$

Where, A_C_ = control of the absorbance and A_S_ = sample of the absorbance.

### Antiproliferative activity

The antiproliferative potential of the *T. toreumaticus* test and spine ethanolic extract was evaluated using a yeast cell model, as described [35]. To prepare the inoculum, 100 mL of sterilized nutrient broth was supplemented with 5 g of commercially available yeast and incubated at 37 °C for 24 h. A cell suspension containing approximately 25.4 × 10⁴ cells was obtained by diluting 1 mL of this seeded broth with 10 mL of sterile distilled water [36]. For each treatment, 1 mL of the yeast inoculum was mixed with varying concentrations of the *T. toreumaticus* extract (50, 100, 150, 200, and 250 µg/mL) along with 2.5 mL of potato dextrose broth (PDB). The control group consisted of yeast inoculum combined only with PDB. All tubes were incubated at 37 °C for 24 h. Post-incubation, cell viability was assessed by staining the yeast suspension with 0.1% methylene blue and examining the cells under a low-power microscope. Transparent (viable) and blue-stained (non-viable) cells were counted across 16 hemocytometer chambers. The mean cell count (cells/mL) and percentage of cell viability were then calculated using standard formulae.


$$\text{Viable}\;\text{cells}/\text{mL}=\text{average}\;\text{no}.\;\text{of}\;\text{viable}\;\text{cells}\;\text{in}\;\text{one}\;\text{square}\;\times \text{dilution}\;\text{factor}\;\times {\text{10}}^{\text{4}}$$

Percentage of cell viability=Total viable cells/Total cells ×100

### Molecular docking

Molecular docking simulations were conducted to explore the interactions between the zoochemical identified in *T. toreumaticus* test and spine (ligands) and several target proteins associated with mosquito biology and disease. These targets included juvenile hormone-binding protein (PDB: 5V13), insect acetylcholinesterase (PDB: 1DX4), *Plasmodium falciparum* lactate dehydrogenase (pfLDH, PDB: 1CEQ), and the anti-chikungunya protein (PDB: 3TRK). Protein structures were retrieved from the Protein Data Bank (PDB), while the structures of the ligands were sourced from the PubChem database. Molecular docking was performed using the PyRx 0.8 platform with AutoDock Vina. The docking simulations were carried out with specific grid dimensions for each target protein (5V13: center x = 254.06, center y = 4.28, center z = 364.18; 1DX4: center x = 24.53, center y = 62.42, center z = 10.06; 1CEQ: center x = 27.10, center y = 25.53, center z = 8.91; 3TRK: center x = 13.25, center y = 23.76, center z = 20.78), as determined by the virtual screening method [37]. The 2D and 3D structures of the docked complexes were visualized using PyMOL, BIOVIA Discovery Studio Visualizer, and UCSF Chimera.

### Statistical examination

All experiments were conducted in triplicate (n = 3). Data was analyzed using IBM SPSS Statistics Version 20.0. Larvicidal and pupicidal bioassay results were evaluated using probit analysis with a logarithm base 10 transformation. A significance level of 0.05 was applied using the heterogeneity factor, with the natural response rate set to none. Analysis parameters included a maximum of 20 iterations, a step size limit of 0.1, and default optimality tolerance. Cytotoxicity data were analyzed using one-way ANOVA, followed by Tukey’s HSD post-hoc test. LC_50_, IC_50_, and EC_50_ values, along with 95% confidence intervals, were calculated. Statistical significance was defined as *p* < 0.05, whereas *p* > 0.05 was considered non-significant.

## Results and discussion

### Zoochemicals extraction and screening

This study provides the first GC-MS characterization of ethanolic extracts from the test and spines of the black sea urchin *T. toreumaticus*, revealing 24 zoochemicals dominated by n-hexadecanoic acid. Comprehensive bioassays confirmed larvicidal and pupicidal activity against *A. aegypti*, AChE inhibition, yeast cytotoxicity, alongside docking predicted effects on PfLDH and CHIKV nsP2 protease. These results establish *T. toreumaticus*, previously documented only for hemolytic and antimicrobial properties, as a marine source for natural products targeting vector-borne diseases.

Fig 1B shows histochemical staining corroborated these findings, toluidine blue yielded blue-green reactions indicative of polyphenols, 2,4-dinitrophenylhydrazine (DNPH) produced orange colour diagnostic for terpenoids, and Wagner’s reagent gave reddish brown coloration confirming alkaloids [22,23,30]. FT-IR spectra of *T. toreumaticus* extracts showed characteristic bands for alcohols (3200–3600 cm^-1^), phenols/aromatics (1400–1600 cm^-1^), amines (1000–1300 cm^-1^), and alkyl halides (600–800 cm^-1^), consistent with terpenoids, alkaloids, and phenolics identified (Fig 1C). These rapid qualitative methods validated secondary metabolite presence in dried sea urchin test/spine powder, aligning with prior FTIR detection of similar functional groups in *T. toreumaticus* aqueous extracts and supporting their role in observed bioactivities.

GC–MS analysis identified 24 zoochemicals in the ethanolic extracts of *T. toreumaticus* test and spines material, including 9-octadecenoic acid, n-hexadecanoic acid, 1-tetradecanol, tetradecanoic acid, and octadecanoic acid (Table 1, Fig 1D). These fatty acids and alcohols align with secondary metabolite profiles documented in other marine species, such as the sea urchins *Salmacis virgulata* and *Diadema setosum*, the Nile crab *Potamonautes niloticus*, and the swimming crab *Charybdis natator*, as well as the marine sponge *Axinella sinoxea*, Sydney rock oyster *Saccostrea glomerata*, and even terrestrial sources like praying mantis *Miomantis paykullii* and *Sunda porcupine* quills (Table 2) [38–45].

**Table 1:** GC-MS characterization of bioactive zoochemicals from the ethanolic extract t. *toreumaticus* ethanolic extract.

Peak	Ret. Index *	Area %	Height %	M. Weight (g/mol)	Molecular formula	Tentative identified Zoochemicals
1	1620	0.86	1.91	224	C_16_H_32_	3-Hexadecene
2	1656	1.22	2.57	214	C_14_H_30_O	1-Tetradecanol
3	1421	2.39	4.48	196	C_14_H_28_	5-Tetradecene
4	4395	1.01	1.29	618	C_44_H_90_	Tetratetracontane
5	2080	0.57	0.62	310	C_22_H_46_	Eicosane, 2,4-dimethyl
6	1769	1.81	1.24	228	C_14_H_28_O_2_	Tetradecanoic acid
7	1900	1.42	2.60	266	C_19_H_38_	1-Nonadecene
8	2009	1.15	2.32	282	C_20_H_42_	Eicosane
9	1320	1.64	3.03	212	C_15_H_32_	Dodecane, 2,6,11-trimethyl
10	–	7.36	5.66	–	–	Unknown
11	1810	1.86	2.34	254	C_18_H_38_	Octadecane
12	1846	1.01	1.72	268	C_19_H_40_	Octadecane, 3-methyl
13		9.64	4.68	–	–	Unknown
14	–	1.30	1.30	398	C_21_H_14_FEN_2_O_3_	Iron, tricarbonyl[N-(phenyl-2-pyridinylmethylene)benzenamine-N,N’]
15	1852	0.85	1.15	296	C_21_H_44_	Heptadecane, 2,6,10,15-tetramethyl
16	1818	1.38	2.06	252	C_18_H_36_	9-Octadecene
17		0.92	1.50	–	–	Unknown
18	2175	2.37	2.22	282	C_18_H_34_O_2_	9-Octadecenoic acid (Z)- (CAS) Oleic acid
19	1968	18.85	16.35	256	C_16_H_32_O_2_	n-Hexadecanoic acid
20	1755	1.50	2.44	228	C_15_H_32_O	1-Pentadecanol
21	1403	0.80	1.09	196	C_14_H_28_	1-Tetradecene
22	1818	1.16	1.10	252	C_18_H_36_	3-Octadecene, (E)-
23	2167	1.63	1.23	284	C_18_H_36_O_2_	Octadecanoic acid (CAS) Stearic acid
24	2153	6.44	6.19	284	C_19_H_40_O	n-Nonadecanol-1
25	–	2.41	2.69	243	C_12_H_10_FN_5_	1H-Purin-6-amine, [(2-fluorophenyl)methyl]
26	1808	8.20	6.54	238	C_16_H_30_O	9-Hexadecenal
27	4765	6.92	6.99	652	C_38_H_68_O_8_	l-(+)-Ascorbic acid 2,6-dihexadecanoate
28	–	0.77	1.31	–	–	Unknown
29	–	0.73	1.12	–	–	Unknown
30	–	11.83	10.24	–	–	Unknown

(*) Ret. Index from Wiley and NIST standard literature databases (NIST05.LIB, NIST05s.LIB, NIST11.lib, NIST11s.lib, WILEY7.LIB).

**Table 2:** Previously reported zoochemicals from different animal sources and marine derivatives.

Zoochemicals	Literature	
	Source	References
9-Octadecenoic acid	Animal (Marine sponge *Axinella sinoxea*), Nile crab *Potamonautes niloticus*.	[38,39]
n-Hexadecanoic acid	Animal (*S. virgulata* and *D. setosum* sea urchin), *Charybdis natator* crabs, Sydney rock oyster *Saccostrea glomerata*	[40–42];
1-Tetradecanol	Animal (*Miomantis paykullii*)	[43]
Tetradecanoic acid	Animal (*Diadema setosum* sea urchin)	[44]
Eicosane	*Sunda Porcupine* Quills	[45]
Octadecanoic acid	Nile crab *Potamonautes niloticus*. Sydney rock oyster *Saccostrea glomerata*, *Sunda Porcupine* Quills	[39,42,45];
1H-Purin-6-amine, [(2-fluorophenyl)methyl]	Earthworm *Pheretima javanica*	[46]
l-(+)-Ascorbic acid 2,6-dihexadecanoate	Sydney rock oyster *Saccostrea glomerata*, *Sunda Porcupine* Quills	[42,45]

n-Hexadecanoic acid (C_16_H_32_O_2_; palmitic acid) emerged as the predominant compound, accounting for 18.85% relative abundance with a peak area of 16.35%. This fatty acid has documented bioactivities, including larvicidal and pupicidal effects against mosquito vectors, repellent properties, and inhibition of acetylcholinesterase, which disrupts insect neurotransmission [45–49]. Such findings suggest that n-hexadecanoic acid contributes substantially to the observed larvicidal and pupicidal efficacy of the *T. toreumaticus* extract in both *in vitro* and *in vivo* assays. The presence of these zoochemicals in marine invertebrates often reflects de novo biosynthesis, dietary uptake, or microbial symbioses, as seen in sponge and echinoderm extracts analyzed by GC–MS. Non-endogenous compounds may also arise from environmental bioaccumulation, though symbiotic microorganisms likely play a key role in generating bioactive fatty acids within *T. toreumaticus*.

### Larvicidal, pupicidal, and AChE inhibitory activity

**Fig 2:**
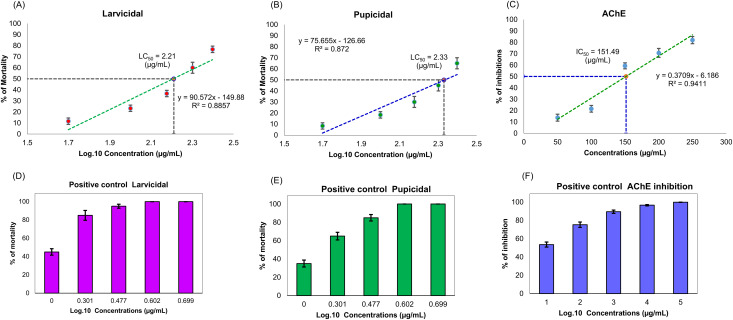
Anti-Mosquito activity of *T. toreumaticus* ethanolic extract against *A. aegypti.* **(A, D)** larvicidal activity, **(B, E)** pupicidal activity, **(C, F)** AChE inhibitory activity.

The ethanolic extract of *T. toreumaticus* showed marked larvicidal activity against 4^th^ instar larvae of *A. aegypti* (LC₅₀ = 164.185 µg/mL) and pupicidal activity against pupae (LC₅₀ = 209.918 µg/mL). In parallel, the extract inhibited acetylcholinesterase (AChE) *in vitro* (IC₅₀ = 151.49 µg/mL), consistent with dose-dependent responses for all bioassays (R² = 0.89, 0.87, and 0.94, respectively; Table 3, Fig 2A–C). AChE inhibition disrupts cholinergic neurotransmission in insects, aligning with the observed larval toxicity and supporting the extract’s potential against mosquito vectors of malaria and other diseases.

**Table 3:** Larvicidal and Pupicidal activity of the ethanolic extract of *T. toreumaticus* against 4^th^ instar *A. aegypti.*

Anti-mosquito target	Lethal concentration (µg/mL)		R^2^	Chi-Square
	LC_50_ (LCL–UCL)	LC_90_ (LCL–UCL)		
Larvicidal	164.185 (130.963–219.286)	474.608 (314.538–1289.606	0.89	X^2^= 1.805; df = 3; Sig. = 0.614
Pupicidal	209.918 (163.619–338.848)	669.204 (389.604–3245.148)	0.87	X^2^= 1.110; df = 3; Sig. = 0.775

LCL: 95% Lower Confidence Limits; UCL: 95% Upper Confidence Limits; a: Since the significance level is greater than.050, no heterogeneity factor is used in the calculation of confidence limits.

Malathion, used as the positive control, displayed superior potency with LC₅₀ values of 1.091 µg/mL (larvae) and 1.386 µg/mL (pupae), also in a concentration-dependent manner (Fig 2D–F). These results are comparable to prior reports of malathion efficacy against *A. aegypti*, where LC₅₀ values ranged from 0.54 µg/mL (laboratory strains) to 3.31 µg/mL (field strains) [50].

**Fig 3:**
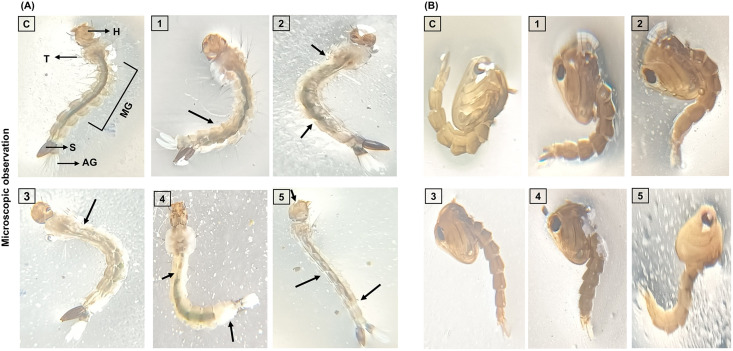
Morphological alterations of 4^th^ instar *A. aegypti* larvae and pupae treated with *T. toreumaticus* extract. **(A)** Larvae mid cut alteration and outer layer damage after 24 h exposure to different concentrations 50-250 µg/ml (arrows indicate outer cuticle damage). **(B)** Pupa morphological changes. (H: Head; T: Thorax: MD: Midgut; AG: Anal gills; S: Siphon).

Treatment of *A. aegypti* 4th instar larvae with *T. toreumaticus* ethanolic extract (50–250 µg/mL, 24 h) induced distinct morphological changes, including midgut disruptions and cuticular damage (Fig 3A and B). Larvae exhibited severe epithelial disorganization, midgut degradation, and impairments to external structures such as anal gills, ventral brush, and siphon, with effects intensifying at higher concentrations relative to untreated controls. Pupae exposed to the same concentrations showed comparable architectural deformities, suggestive of disrupted ecdysial processes and developmental arrest. The dose-dependent structural impairments in both stages, consistent with toxicity mechanisms involving integumental penetration and midgut cytotoxicity reported in botanical larvicides.

The larvicidal potency of *T. toreumaticus* ethanolic extract (LC₅₀ = 164.185 µg/mL against *A. aegypti* 4^th^ instar larvae) compares favorably to various plant-derived larvicides reported in the literature. For instance, methanolic and aqueous extracts of *Acacia nilotica* achieved 50% mortality in *Culex pipiens* larvae at LC₅₀ values of 200.50 and 256.40 µg/mL, respectively [51]. Similarly, ethanolic extract of *Artemisia absinthium* yielded an LC_50_ of 694.3 µg/mL against *A. aegypti*, while Ajaegbu et al. documented a broad range of LC₅₀ values (412.90–17,640.41 µg/mL) across 15 plant extracts tested on the same species [52]. These benchmarks highlight the competitive efficacy of the *T. toreumaticus* zoo-extract, which outperforms several botanical counterparts in potency against *A. aegypti* while matching or exceeding activities seen in coastal plant extracts like *Spinifex littoreus* (LC₅₀ = 67.058 ppm). Such performance underscores the value of marine invertebrate sources for developing natural vector control agents, particularly given their fatty acid profiles that enhance integumental penetration and midgut disruption.

**Fig 4:**
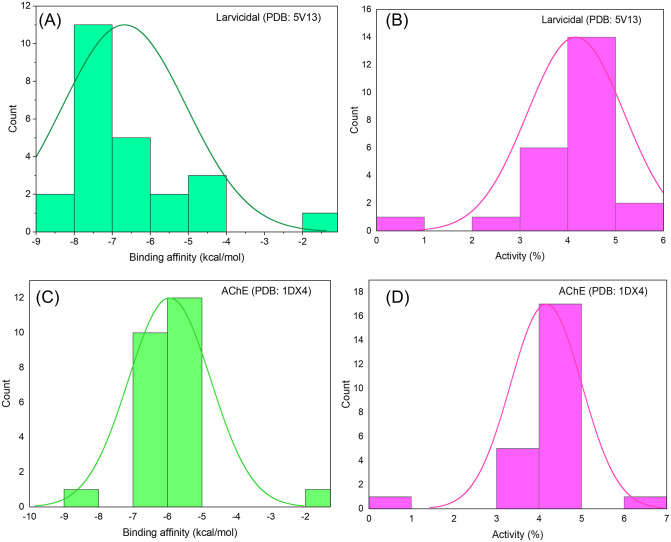
Binding affinity and predicted activity of *T. toreumaticus* zoochemicals against molecular target. **(A-B)** Larvicidal (PDB: 5V13), **(C-D)** AChE inhibition (PDB: 1DX4).

**Fig 5:**
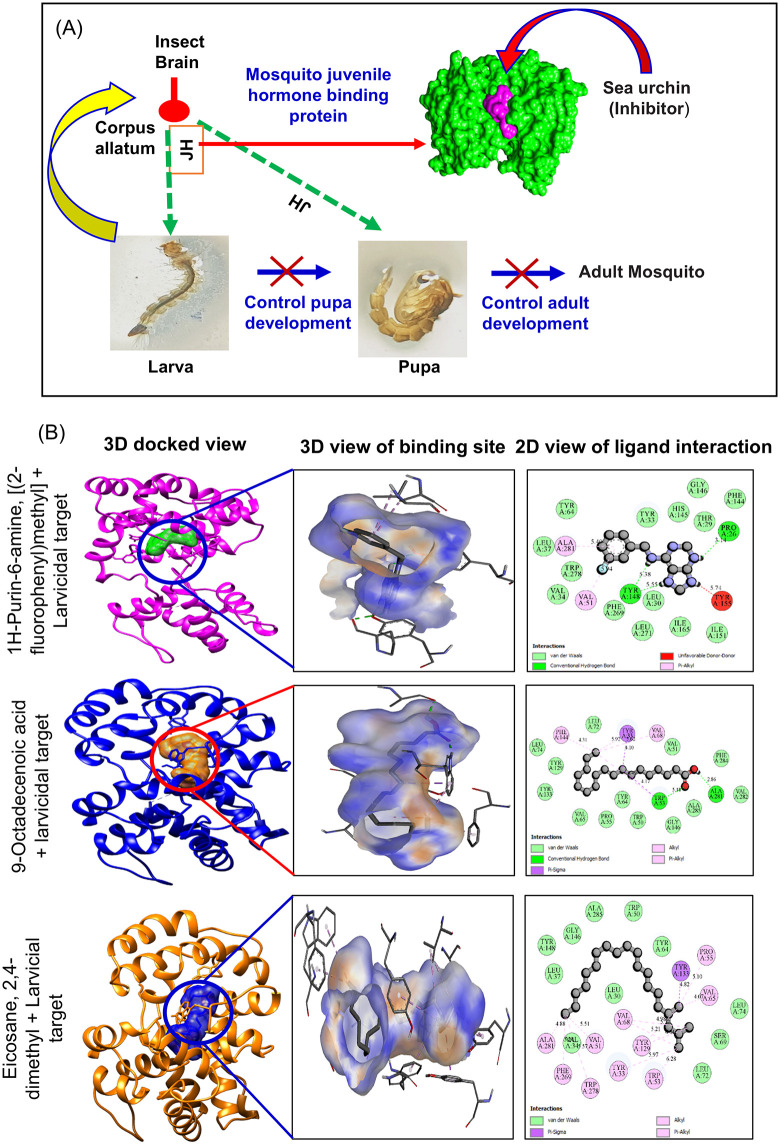
Larval growth regulating activity of *T. toreumaticus* zoochemicals. **(A)** *Proposed role of T. toreumaticus* zoochemicals as larvae growth regulator for insect control. **(B)** 3D and 2D Molecular docking of zoochemicals against mosquito growth regulators of juvenile hormone binding protein (PDB: 5V13).

*In silico* docking studies validated the larvicidal potential of *T. toreumaticus* zoochemicals, revealing strong binding affinities to mosquito juvenile hormone binding protein (PDB: 5V13) and acetylcholinesterase (AChE; PDB: 1DX4), key targets for disrupting endocrine regulation and cholinergic neurotransmission, respectively (Table 4). Larvicidal compounds predominantly showed binding energies of –8 to –7 kcal/mol activity percentages of 4–5% (Fig 4A and B), while AChE inhibitors clustered at –6 to –5 kcal/mol and similar activity levels (Fig 4C and D). These affinities align with reported thresholds for bioactive ligands against insect targets, where values below –7 kcal/mol indicate favorable hydrophobic and hydrogen bonding interactions within active sites. A schematic of the proposed mechanism, fatty acid-mediated disruption of hormone signaling and AChE inhibition leading to larval developmental arrest, is depicted in Fig 5A, with 2D/3D interaction maps for top ligands in Fig 5B.

**Table 4:** *In silico* analysis of larvicidal and anti-malarial activity of identified zoochemicals from *T. toreumaticus.*

Zoochemicals	Larvicidal (PDB: 5V13)		AChE (PDB: 1DX4)		Anti-malarial (PDB: 1CEQ)		Anti-chikungunya (PDB: 3TRK)	
	kcal/mol	Activity (%)	kcal/mol	Activity (%)	kcal/mol	Activity (%)	kcal/mol	Activity (%)
3-Hexadecene	−7.7	4.803	−5.9	4.140	−4.5	3.978	−4.3	3.668
1-Tetradecanol	−6.7	4.179	−6	4.210	−4.1	3.625	−4.3	3.668
5-Tetradecene	−7.3	4.553	−5.8	4.070	−4.9	4.332	−4.8	4.095
Tetratetracontane	−4.9	3.056	−6.5	4.561	−4.8	4.244	−5.1	4.351
Eicosane, 2,4-dimethyl	−8.1	5.053	−5.6	3.929	−4.8	4.244	−5.6	4.778
Tetradecanoic acid	−7.3	4.553	−6	4.210	−4.9	4.332	−5.2	4.436
1-Nonadecene	−4.7	2.932	−6.3	4.421	−4.7	4.155	−4.7	4.010
Eicosane	−5.2	3.243	−6.1	4.280	−4.6	4.067	−5.3	4.522
Dodecane, 2,6,11-trimethyl	−7.9	4.928	−5.4	3.789	−4.5	3.978	−5	4.266
Octadecane	−7.5	4.678	−5.9	4.140	−4.5	3.978	−5	4.266
Octadecane, 3-methyl	−7.9	4.928	−6	4.210	−4.7	4.155	−4.7	4.010
Iron, tricarbonyl[N-(phenyl-2-pyridinylmethylene)benzenamine-N,N’]	−1.2	0.748	−1.4	0.982	−1.3	1.149	−1.2	1.023
Heptadecane, 2,6,10,15-tetramethyl	−5	3.119	−6	4.210	−4.8	4.244	−5.4	4.607
9-Octadecene	−7.9	4.928	−6.3	4.421	−4.8	4.244	−5.1	4.351
9-Octadecenoic acid	−8	4.990	−6.6	4.631	−5.4	4.774	−4.8	4.095
n-Hexadecanoic acid	−7.5	4.678	−6.4	4.491	−4.9	4.332	−5	4.266
1-Pentadecanol	−6.9	4.304	−6.1	4.280	−4.5	3.978	−4.3	3.668
1-Tetradecene	−6.8	4.242	−5.5	3.859	−4.7	4.155	−4.3	3.668
3-Octadecene	−7.9	4.928	−5.4	3.789	−4.8	4.244	−5	4.266
Octadecanoic acid	−5.6	3.493	−6.8	4.771	−5.3	4.686	−5	4.266
n-Nonadecanol-1	−6.1	3.805	−6.1	4.280	−4.5	3.978	−4.4	3.754
1H-Purin-6-amine, [(2-fluorophenyl)methyl]	−8.6	5.364	−8.9	6.245	−6.7	5.923	−7.3	6.228
9-Hexadecenal	−7.4	4.616	−5.2	3.649	−4.3	3.801	−4.9	4.180
l-(+)-Ascorbic acid 2,6-dihexadecanoate	−6.2	3.867	−6.3	4.421	−6.1	5.393	−6.5	5.546

In silico molecular docking Activity (%) = C/ ∑C× 100. Where C is the single compound binding affinity (kcal/mol); ∑_C_ is the sum of the compound binding affinity (kcal/mol); 100 is the reference percentage (%).

Zoochemicals from marine animal extracts and phytochemicals from plants exhibit comparable larvicidal activities, driven by overlapping secondary metabolites such as fatty acids, terpenoids, and alkaloids that target mosquito physiology through integumental disruption, midgut toxicity, and enzyme inhibition. For example, zinc oxide nanoparticles biosynthesized using the marine sponge *Spongia officinalis* demonstrated potent larvicidal effects against *Culex pipiens* and *Anopheles pharoensis* larvae, highlighting the efficacy of animal-derived materials [53]. Plant extracts show similar promise: methanolic fractions often synergize to enhance mortality via AChE inhibition and developmental arrest, while the aqueous extract of *Ocimum americanum* impairs mosquito reproduction, reducing vector competence for diseases like dengue [54]. These parallels position *T. toreumaticus* ethanolic extract as a viable marine alternative to botanical larvicides, with n-hexadecanoic acid contributing to both potency and eco-compatibility in integrated mosquito management.

Juvenile hormone (JH) regulates mosquito larval development by preventing premature metamorphosis, maintaining larval characteristics until the final instar; its disruption induces developmental arrest and mortality, making JH-binding proteins (PDB: 5V13) prime targets for vector control agents [55]. Bioinformatics and docking studies have identified JH receptor inhibitors effective against *A. aegypti*, with binding affinities below –7 kcal/mol, predicting strong disruption of endocrine signalling [56–58]. Zoochemicals from *T. toreumaticus* exhibited comparable inhibitory interactions with the *A. aegypti* JH-binding protein (PDB: 5V13), validating their role in the observed larvicidal effects through interference with hormone titer regulation and ecdysis. This mechanism complements AChE inhibition, targeting complementary pathways, endocrine and cholinergic, for enhanced mosquito control efficacy.

**Fig 6:**
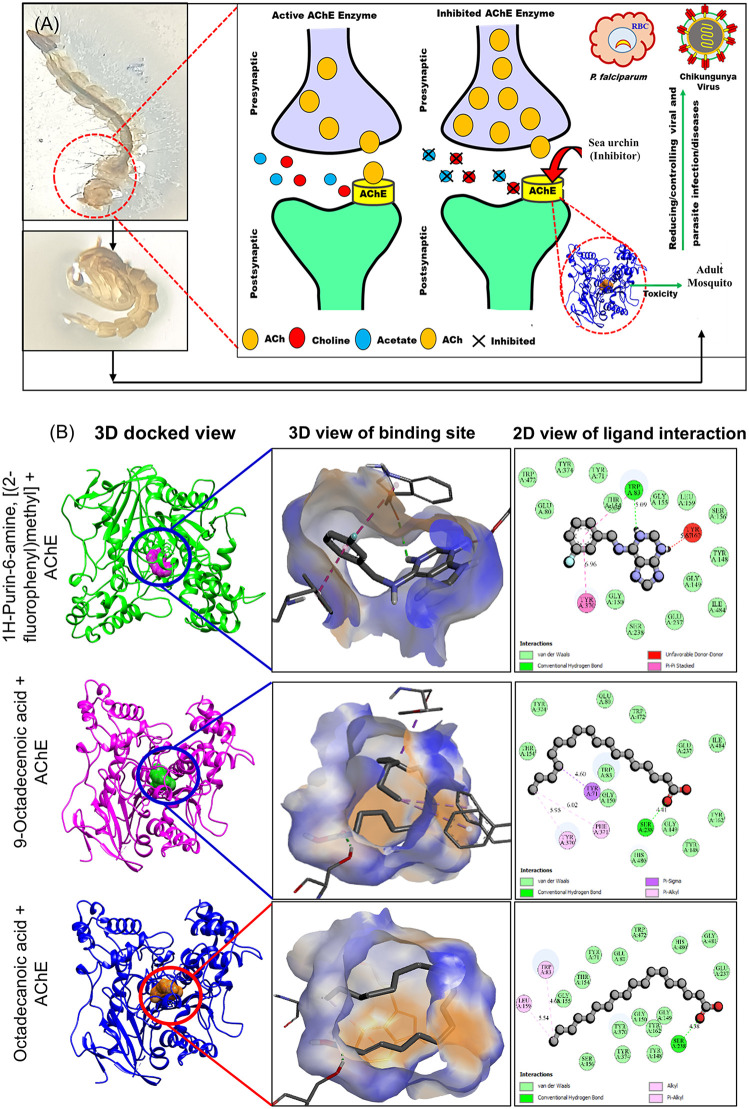
Inhibitory action of *T. toreumaticus* zoochemicals against AChE. **(A)** Proposed inhibitory mechanism of sea urchin *T. toreumaticus* on *A. aegypti* larvae AChE enzyme. **(B)** 3D and 2D Molecular docking view of zoochemicals against AChE (PDB: 1DX4).

AChE hydrolyzes acetylcholine to terminate nerve impulses in insects; its inhibition causes neurotransmitter accumulation, cholinergic overstimulation, and larval paralysis or death, establishing it as a validated insecticide target [59,60]. The *T. toreumaticus* ethanolic extract inhibited *A. aegypti* AChE in vitro (IC₅₀ = 151.49 µg/mL), consistent with dose-dependent larval toxicity and midgut histopathology observed earlier. This mechanism parallels AChE inhibition by natural extracts in *Culex pipiens* and sea urchin *Salmacis virgulata* against *T. castaneum*, as well as actinomycete metabolites [6,32]. Docking simulations using the Drosophila AChE template (PDB: 1DX4) confirmed strong binding by key zoochemicals (Fig 6A and B), with interaction maps highlighting hydrophobic and H-bond contacts in the active site gorge [61]. By targeting both JH signaling and AChE, the extract offers dual-mode bioactivity comparable to synthetic organophosphates but with marine-derived selectivity, positioning it as a candidate for sustainable *A. aegypti* control and reducing vector-borne disease transmission.

### *In silico* anti-malarial activity

*Plasmodium falciparum*, the protozoan parasite responsible for malaria, undergoes a complex life cycle involving development in both human and mosquito hosts. A critical enzyme for the parasite’s survival during the blood stage is *P. falciparum* lactate dehydrogenase (PfLDH), which catalyzes the conversion of pyruvate to lactate while regenerating NAD+, thereby sustaining high rates of glycolysis essential for energy production under anaerobic conditions. Due to its indispensable role, PfLDH is a promising target for antimalarial drug development. Structurally, PfLDH possesses unique features, including an insertion in the substrate specificity loop and distinct active site pockets, which differentiate it from human LDH and facilitate selective inhibitor design targeting the NADH and substrate-binding sites (PDB: 1CEQ). This enzyme’s activity is essential for maintaining the parasite’s metabolic processes during the febrile episodes characteristic of malaria, and inhibitors that disrupt PfLDH function can impair parasite survival, making PfLDH a focal point for developing novel therapeutics with specificity to the parasite enzyme to minimize host toxicity [62,63].

**Fig 7:**
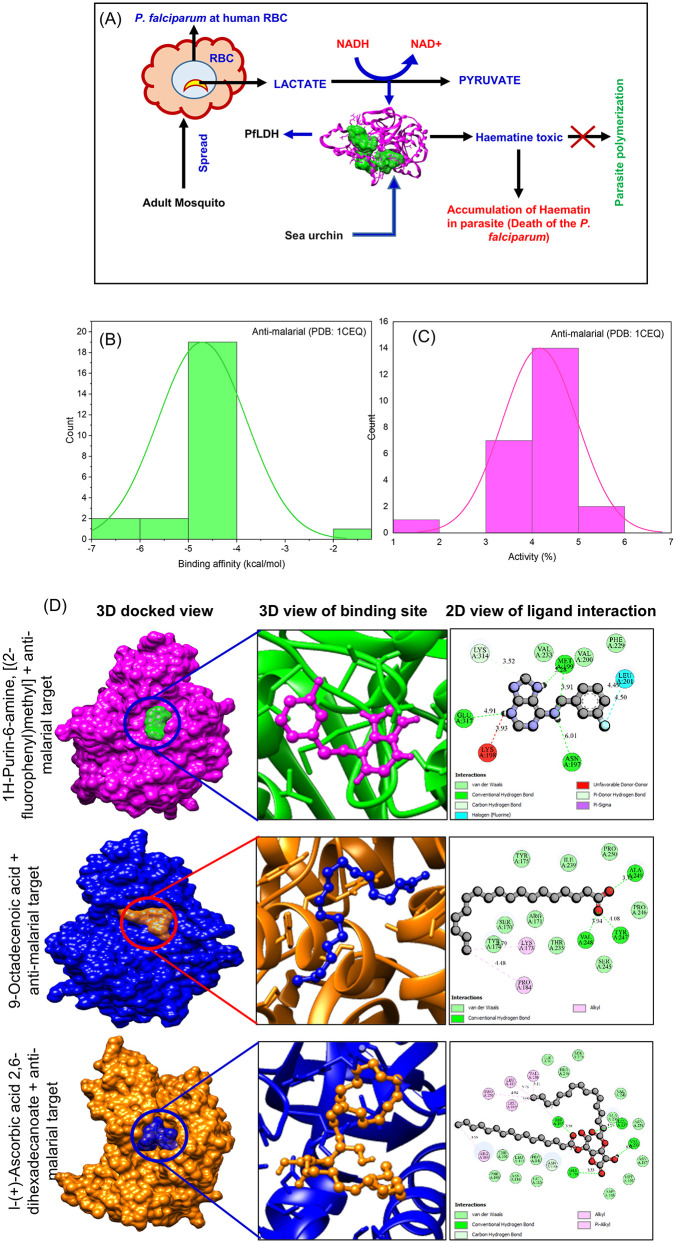
Anti-malarial potential of *T. toreumaticus* against *Plasmodium falciparum* lactate dehydrogenase enzyme (pfLDH). **(A)** Mode of inhibitory action of sea urchin *T. toreumaticus* against pfLDH (PDB: 1CEQ). **(B-C)** Histogram plot of zoochemicals binding affinity (Kcal/mol) and activity (%) against the molecular target (PDB: 1CEQ). **(D)** 3D and 2D Molecular docking of zoochemicals against *pf*LDH (PDB: 1CEQ).

Molecular docking analysis evaluated the *T. toreumaticus* zoochemicals against *P. falciparum* lactate dehydrogenase (PfLDH; PDB: 1CEQ), a terminal glycolytic enzyme critical for NAD^+^ regeneration and parasite survival in erythrocytes (Table 4). Several compounds exhibited favorable binding affinities of –5 to –4 kcal/mol (Fig 7A and B), with activity percentages of 4–5% (Fig 7C), indicating competitive inhibition at the NADH cofactor and substrate-binding sites. This disruption blocks pyruvate-to-lactate conversion, halting anaerobic ATP production and arresting intra-erythrocytic development, a validated antimalarial mechanism. The schematic in Fig 7D illustrates fatty acid-mediated active site occlusion, akin to known PfLDH inhibitors [62,64]. Terpenoids and alkaloids among the identified zoochemicals align with documented marine natural products targeting PfLDH, positioning *T. toreumaticus* as a promising source for selective antimalarials with dual larvicidal and parasiticidal potential.

### *In silico* Anti-chikungunya activity

**Fig 8:**
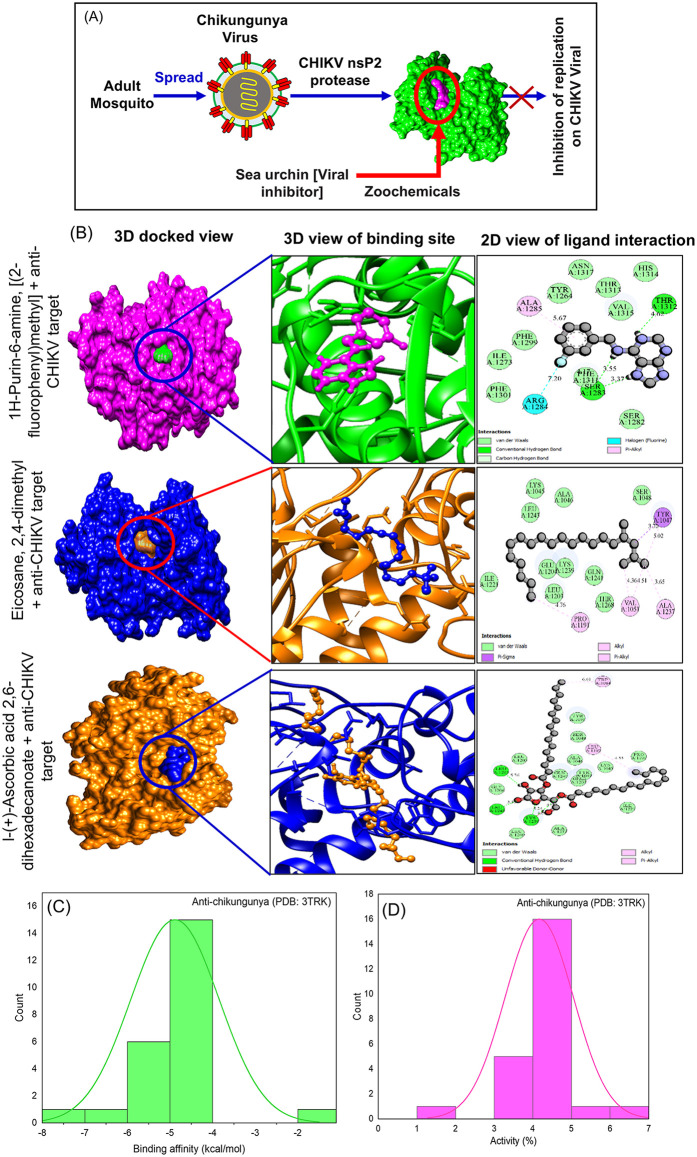
Anti-chikungunya activity of *T. toreumaticus* zoochemicals against CHIKV nsP2 protease. **(A)** Mode of action of *T. toreumaticus* zoochemicals inhibitory activity against Chikungunya virus’s NsP2 protease. **(B)** 3D and 2D Molecular docking of zoochemicals against the CHIKV nsP2 protease (PDB: 3TRK). **(C-D)**. Histogram plot of zoochemicals binding affinity (Kcal/mol) and activity (%) against molecular target (PDB: 3TRK).

Chikungunya virus (CHIKV), transmitted by Aedes mosquitoes, causes debilitating arthritic disease prevalent in Asia and Africa; its non-structural protein 2 (nsP2) protease cleaves the viral polyprotein precursor (nsP1234) at specific junctions (e.g., AGA/GII, AGC/APS), enabling replicase complex formation essential for RNA replication [65,66]. Docking analysis of *T. toreumaticus* zoochemicals against CHIKV nsP2 protease (PDB: 3TRK) revealed competitive binding affinities of –5 to –4 kcal/mol for most compounds (Table 4; Fig 8A–C), with activity percentages of 4–5% (Fig 8D), targeting the papain-like active site (Cys478-His548 dyad) and blocking substrate access via flexible loops [67]. This inhibition disrupts polyprotein processing, halting genomic and subgenomic RNA synthesis, akin to known phenolic and thiazolbenzamide inhibitors. These marine-derived leads align with strategies exploiting nsP2’s multifunctional helicase/protease domains for broad alphavirus control, positioning *T. toreumaticus* zoochemicals as dual action candidates against CHIKV replication and its Aedes vectors.

### *In vitro* anti-proliferative activity (Cytotoxicity)

**Fig 9:**
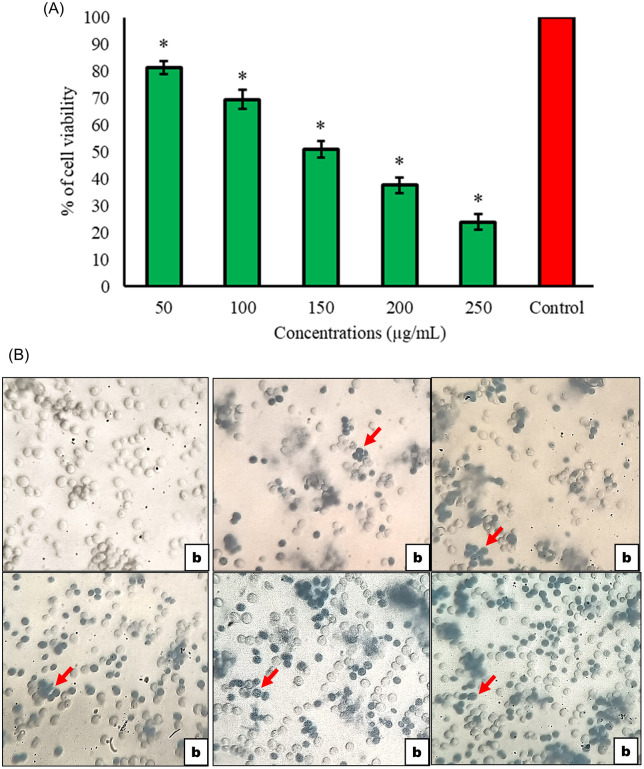
Anti-proliferative activity of *T. toreumaticus* extract against yeast cells. **(A)** *In vitro* anti-proliferative activity of *T. toreumaticus* ethanolic extract. (*p < 0.05 vs control; one-way ANOVA). **(B)** Morphology changes in yeast cells with treatment of sea urchin *T. toreumaticus* ethanolic extract: (a) Control; (b – f) different concentrations of *T. toreumaticus* ethanolic extract. Red arrow or blue colour cells indicate dead cells.

The *T. toreumaticus* ethanolic extract exhibited potent cytotoxicity toward *Saccharomyces cerevisiae*, achieving an EC₅₀ of 159.27 µg/mL in a dose-dependent manner (R² = 0.99), with 76.05 ± 3.01% cell death at 250 µg/mL versus untreated controls (Fig 9A). Light microscopy confirmed non-viable cells via blue staining, consistent with resazurin or trypan blue assays standard for yeast viability assessment (Fig 9B) [66]. This profile parallels antimalarials like proguanil-atovaquone, which collapse mitochondrial membrane potential in yeast, and artemisinin derivatives that generate ROS-mediated growth arrest [68,69]. Such cross activity in *S. cerevisiae*, a validated eukaryotic model for screening antimalarials due to conserved glycolytic and mitochondrial targets, supports the extract’s zoochemicals as multi-target leads bridging larvicidal, antimalarial, and cytotoxic mechanisms.

### Statistical modeling of sea urchin *T. toreumaticus in vitro* and *silico* efficacy

Correlation matrices (Tables 5, 6) demonstrate strong congruence between *in vivo, in vitro*, and in silico results for the *T. toreumaticus* ethanolic extract, validating assay consistency. *In vivo* and *in vitro* data showed high Pearson correlations (r = 0.944–0.994) across larvicidal potency, AChE inhibition (IC₅₀), and yeast cytotoxicity (EC₅₀), confirming reproducible biological activity spanning endocrine disruption, neurotoxicity, and antiproliferative effects. In silico docking scores correlated moderately to strongly (r = 0.592–0.934) with experimental endpoints, linking zoochemical binding affinities at JHBP (PDB: 5V13), AChE (PDB: 1DX4), PfLDH (PDB: 1CEQ), and CHIKV nsP2 (PDB: 3TRK) to observed bioefficacy and supporting structure activity predictions.

**Table 5:** *In vitro* study correlation matrix (percentage and efficacy) of zoo-extract from *T. toreumaticus.*

Assay	Larvicidal (%)	Pupicidal (%)	AChE inhibition (%)	Yeast cell growth inhibition (%)
Larvicidal (%)	1			
Pupicidal (%)	0.994	1		
AChE inhibition (%)	0.952	0.944	1	
Yeast cell growth inhibition (%)	0.989	0.986	0.982	1

(N=5; Concentration 50 to 250µg/mL).

**Table 6:** *In silico* study correlation matrix of molecular docking Binding affinity (Kcal/mol) of zoochemicals from *T. toreumaticus.*

Molecular target	Larvicidal (PDB: 5V13)	AChE (PDB: 1DX4)	Anti-malarial (PDB: 1CEQ)	Anti-chikungunya (PDB: 3TRK)
Larvicidal (PDB: 5V13)	1			
AChE (PDB: 1DX4)	0.592	1		
Anti-malarial (PDB: 1CEQ)	0.636	0.921	1	
Anti-chikungunya (PDB: 3TRK)	0.602	0.854	0.934	1

(N = 24).

The highest correlation coefficients linked AChE inhibition to antimalarial (PfLDH) and anti-CHIKV (nsP2) activities (r = 1 > 0.80), indicating that *T. toreumaticus* zoochemicals, particularly fatty acids like n-hexadecanoic acid, exhibit multitarget binding across cholinergic, glycolytic, and proteolytic pathways in mosquitoes, protozoa, and alphaviruses. Moderate correlations between larvicidal potency and other endpoints (r = 0.65 > 0.59) reflect the extract’s broad-spectrum profile, where endocrine disruption synergizes with neurotoxicity for enhanced vector control [70]. These integrated *in vivo, in vitro*, and in silico correlations validate docking predictions for multifunctional marine leads, supporting the development of *T. toreumaticus* derived agents for integrated management of malaria and chikungunya transmission.

This study demonstrates the biotechnological potential of marine zoowaste, including sea urchin tests and spines, mollusk/arthropod shells, sponges, and fish processing residues as sustainable sources of bioactive zoochemicals for larvicidal, antimalarial, and antiviral applications. *T. toreumaticus* extracts exemplify circular economy principles by converting fishery discards into high-value insecticidal agents, reducing coastal pollution while providing eco-friendly alternatives to synthetic pesticides. Such approaches align with blue biotechnology strategies that integrate waste biorefinery with vector control, promoting ecological restoration alongside biomedical innovation from underutilized marine biomass.

## Conclusion

The ethanolic extract of *T. toreumaticus*, including its test and spines, demonstrated significant biological potential. FT-IR analysis revealed the presence of key functional groups, including alcohols, phenols, amines, aromatics, and halides, corresponding to bioactive classes such as phenols, alkaloids, and terpenoids. Histo-zoochemical analysis further provided a rapid and preliminary confirmation of these major secondary zoochemicals. GC-MS profiling identified 24 distinct zoochemical compounds, several of which are known for their insecticidal and cytotoxic properties.

The larvicidal activity of *T. toreumaticus* extract is particularly notable, correlating with the high concentration of n-hexadecanoic acid, which has documented insecticidal activity and exhibits strong binding affinity toward molecular targets associated with larvicidal, acetylcholinesterase inhibitory, antiviral, and antimalarial effects. These findings suggest that *T. toreumaticus* represents a promising natural alternative to synthetic insecticides and antimicrobial agents, with potential applications in controlling mosquito vectors and microbial pathogens. Overall, the present zoo-ethanolic extract of *T. toreumaticus* demonstrates effective control over mosquito metamorphic stages (larvae and pupae) and shows comparable efficacy to plant-derived insecticides, supporting its potential as a novel, eco-friendly bioinsecticide.
